# Application of the path-repairing technique and virus optimization algorithm for the dimensional synthesis of four-bar mechanisms

**DOI:** 10.1007/s43452-023-00670-2

**Published:** 2023-04-30

**Authors:** Jakub Krzysztof Grabski, Martyna Sopa, Agata Mrozek

**Affiliations:** Institute of Applied Mechanics, Faculty of Mechanical Engineering, Poznan University of Technology, ul. Jana Pawła II 24, 60-965 Poznań, Poland

**Keywords:** Nature-inspired algorithms, Metaheuristics, Virus optimization algorithm, Four-bar mechanism, Dimensional synthesis of mechanisms

## Abstract

This paper considers the synthesis of the four-bar mechanism. It is treated here as an optimization problem, in which an objective function is defined. To solve this problem, a metaheuristic called the virus optimization algorithm is employed. Furthermore, a new path-repairing technique recently published by Sleesongsom and Bureerat is applied instead of the very common technique related to the application of a penalty function. This makes the search by means of the metaheuristic more efficient. Furthermore, the obtained results are very accurate.

## Introduction

The four-bar mechanism is one of the most popular mechanisms used in the real world. It is applied in bicycles, oil well pumps, compressors, locking pliers, and so on. Therefore, this kind of mechanism and its analysis are of primary interest to many studies. In general, in the theory of mechanisms, one can distinguish two main types of kinematic syntheses: dimensional and type syntheses. In the first, there is given a type of mechanism, for example, the four-bar mechanism, and a path, function or motion to generate, and the aim is to find the dimensions. In the second, the task to be performed by the mechanism is known, while the type of mechanism is to be determined without regard to the dimensions. In this study, we consider the dimensional synthesis of the four-bar mechanism.

Recently, nature-inspired algorithms have become more and more popular in science and engineering. These algorithms incorporate, for example, artificial neural networks, fuzzy systems and many others. All these approaches have a wide range of applications in the real world. One special group of nature-inspired algorithms are metaheuristic. They can be successfully applied in many optimization problems. The main advantage of this group of algorithms is that they do not need derivatives to work nor any initial guesses to start the computations. Most often, the set of initial solutions, called the population, is randomly selected. However, the use of metaheuristics does not guarantee finding the optimal solution, and it is difficult to estimate the real time of computations. Furthermore, metaheuristics always have some parameters that are strongly problem dependent. Thus, metaheuristics have no universal parameters which work well for every optimization problem. Therefore, metaheuristics are generally applied to more complicated problems subject to nonlinear constraints, for which the classical methods fail or can find only local optimal solutions, such as solutions that are strongly dependent on the initial guess. Over the years, various researchers have proposed many metaheuristic algorithms. The most common is probably the genetic algorithm [1]. However, others are also very popular in the literature, including simulated annealing [2], ant colony optimization [3] and particle swarm optimization [4]. Currently, more and more new metaheuristics are being proposed in the literature; these are inspired by various natural phenomena, such as bacterial foraging [5], dolphin echolocation [6], forest spreading [7], plant propagation [8], river formation dynamics [9] and the water cycle [10]. Some metaheuristics have already been applied to the synthesis of the four-bar mechanism. In 2002, Zhou and Cheung published a paper on a modified genetic algorithm (GA) for the synthesis of the adjustable four-bar mechanism [11]. In the same year, an application of the GA was proposed by Cabrera et al. [12]. A combination of the GA with fuzzy logic (FL) was applied by Laribi et al. in 2004 [13]. The ant colony optimization (ACO) in combination with a gradient search, called the ant–gradient search method, was proposed by Smaili and Diab for solving this problem in 2007 [14]. In 2015, Ebrahimi and Payvandy applied the imperialist competitive algorithm (ICA) and compared the results with results from other metaheuristic algorithms [15]. Recently, Bureerat and Sleesongsom applied a self-adaptive teaching–learning-based optimization to the synthesis of the four-bar mechanism [16]. In 2022, Qaiyum and Mohammad applied the improved harmony search algorithm [17], while Huang et al. used the repellency evolutionary algorithm [18]. Kang et al. compared various metaheuristic optimization algorithm for path synthesis of four-bar mechanisms [19].

In the era of coronavirus, the metaheuristics inspired by viruses have proven to be very interesting. In 2016, Liang and Cuevas Juarez published a paper [20] in which they proposed a new metaheuristic, called the virus optimization algorithm (VOA), and investigated the results obtained in solving eight benchmark functions. The algorithm was based on the authors’ previous works [21, 22]. Although the original work by Liang and Cuevas Juarez was published recently [20], the VOA has been successfully applied to solve several optimization problems. A multi-objective economic strategy dispatching problem was solved by means of the VOA and the harmony search algorithm by Liang and Juarez [23]. This problem is commonly known in the literature as the combined economic-emission dispatch, and the basic task is to achieve the lowest cost possible in combination with the smallest amount of pollutant. The results obtained by the authors using the harmony search algorithm was only slightly better than those obtained using the VOA, and the computational time of the VOA was much shorter. Omenzetter and Turnbull tried to detect potential damage to a wind turbine blade by application of the finite element method [24]. This numerical method was calibrated using two metaheuristics: the firefly algorithm and the VOA. The results of the application of these two algorithms were compared by the authors. Aungkulanon and Luangpaiboon applied the VOA and elephant swarm water search to predict mortality in Thailand [25]. Better results were obtained using the VOA. Recently, Behnood and collaborators used this algorithm in combination with the adaptive network-based fuzzy inference system to forecast the spread of COVID-19 in the USA based on population density and climate parameters [26]. Recently, Liang and Cuevas Juarez proposed a modified self-adaptive version of the VOA [27]. Also recently, Grabski and Mrozek identified the elastoplastic parameters of rods from torsion tests by means of the VOA in combination with the method of fundamental solutions and radial basis functions [28]. Based on this short literature review, one can see that metaheuristics, including the VOA, have a wide range of applications. The VOA has the advantage of a relatively small number of parameters, which must be adjusted in order to solve the considered problem. However, the VOA has not been used yet for the kinematic synthesis of mechanisms.

In the dimensional synthesis of the four-bar mechanism, a common technique for the satisfaction of the constraints is the application of the penalty function, which indicates that if any constraint is unsatisfied, then the objective function is assigned to a very large value. In this way, in the case of a minimalization problem, the set of decision variables for which such a situation occurred will be never found as the optimal solution. This is a simple approach for handling the nonlinear constraints in the optimization problem. However, this technique is very often ineffective in practice, especially in the case of the synthesis of the four-bar mechanism. In the literature, the penalty function method was proposed by Allzade et al. in 1975 [29]. It is very often used with metaheuristics in the synthesis of four-bar mechanism, for example, with genetic algorithm [12], differential evolution [30, 31], or with the combination of genetic algorithm with differential evolution algorithm [32]. One of the constraints in this analysis is the ascending order of the angles, which, among others, are decision variables and represent subsequent angular positions of the crank. This issue has been noted in the literature over the years. Recently, Sleesongsom and Bureerat proposed the path-repairing technique to avoid this problem [33]. In this approach, all those potential solutions which do not satisfy the ascending order of the angles, or do not satisfy the Grashof criterion, are repairing using two additional algorithms. This paper employs this approach.

In this paper, we apply the path-repairing technique in combination with the VOA for the synthesis of the four-bar mechanism. Furthermore, the path-repairing technique has been proposed for constraint handling. The paper is organized as follows. In Sect. 2, the four-bar mechanism and some important equations, based on which the positions of the bars can be calculated, are described. An objective function and constraints formulation are included in Sect. 3. The path-repairing technique and the VOA are described in Sects. 4 and 5, respectively. The obtained results with comparisons to previous studies are presented in Sect. 6. Finally, Sect. 7 includes conclusions from this study.

## Four-bar mechanism and positions of its bars

**Fig. 1:**
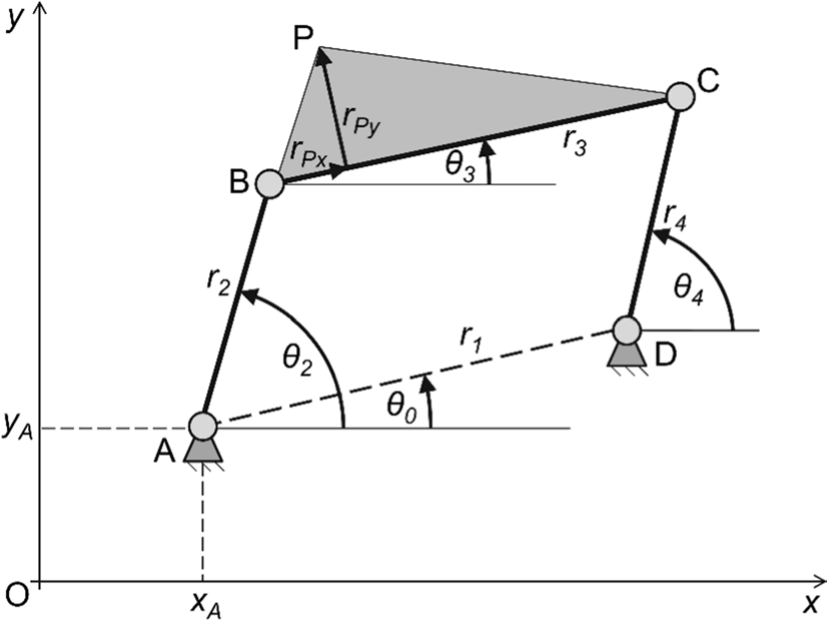
Four-bar mechanism

The four-bar mechanism consists of three movable links and one immovable link, as depicted in Fig. 1. Let us assume that we know the dimensions of the mechanism, and we want to determine the positions of the four-bar mechanism elements, in particular, the position of point $\mathrm{P}$ (see Fig. 1). This can be done using the well-known Freudenstein’s method [34, 35]. The angle ${\theta }_{3}$ can be calculated based on the following formula:

1 $${\theta }_{3}={\theta }_{3a/3b}={\theta }_{0}+2{\mathrm{tan}}^{-1}\left(\frac{-G\pm \sqrt{{G}^{2}-4FH}}{2F}\right),$$

where

2 $$F=\mathrm{cos}\left({\theta }_{2}-{\theta }_{0}\right)-{K}_{1}+{K}_{2}\mathrm{cos}\left({\theta }_{2}-{\theta }_{0}\right)+{K}_{3,}$$

3 $$G=-2\mathrm{sin}\left({\theta }_{2}-{\theta }_{0}\right),$$

4 $$H={K}_{1}+\left({K}_{2}-1\right)\mathrm{cos}\left({\theta }_{2}-{\theta }_{0}\right)+{K}_{3,}$$

5 $${K}_{1}=\frac{{r}_{1}}{{r}_{2}},$$

6 $${K}_{2}=\frac{{r}_{1}}{{r}_{3}},$$

7 $${K}_{3}=\frac{{r}_{4}^{2}-{r}_{1}^{2}-{r}_{2}^{2}-{r}_{3}^{2}}{{2r}_{2}{r}_{3}}.$$

When the angle ${\theta }_{3}$ has already been determined according to Eq. (1) along with Eqs. (2)–(7), the angular positions of all parts of the mechanism are known. Thus, the position $\left({x}_{\mathrm{P}},{y}_{\mathrm{P}}\right)$ of point $\mathrm{P}$ can be obtained from the following relations:

8 $${x}_{\mathrm{P}}={x}_{\mathrm{A}}+{r}_{2}\mathrm{cos}\left({\theta }_{2}\right)+{r}_{\mathrm{P}x}\mathrm{cos}\left({\theta }_{3}\right)-{r}_{\mathrm{P}y}\mathrm{sin}\left({\theta }_{3}\right),$$

9 $${y}_{\mathrm{P}}={{y}_{\mathrm{A}}+r}_{2}\mathrm{sin}\left({\theta }_{2}\right)+{r}_{\mathrm{P}x}\mathrm{sin}\left({\theta }_{3}\right)+{r}_{\mathrm{P}y}\mathrm{cos}\left({\theta }_{3}\right).$$

The above relations are sufficient to calculate the objective function, which is related to controlling the position of point $\mathrm{P}$ and is defined in the next section. However, to obtain the positions of all the four-bar mechanism elements, the angular position of the last link must be calculated. In a similar way as for ${\theta }_{3}$, it can be expressed as:

10 $${\theta }_{4}={\theta }_{4a/4b}={\theta }_{0}+2{\mathrm{tan}}^{-1}\left(\frac{-J\pm \sqrt{{J}^{2}-4IM}}{2I}\right),$$

where

11 $$I=\mathrm{cos}\left({\theta }_{2}-{\theta }_{0}\right)-{K}_{1}-{K}_{4}\mathrm{cos}\left({\theta }_{2}-{\theta }_{0}\right)+{K}_{5},$$

12 $$J=-2\mathrm{sin}\left({\theta }_{2}-{\theta }_{0}\right),$$

13 $$M={K}_{1}-\left({K}_{4}+1\right)\mathrm{cos}\left({\theta }_{2}-{\theta }_{0}\right)+{K}_{5},$$

14 $${K}_{4}=\frac{{r}_{1}}{{r}_{4}},$$

15 $${K}_{5}=\frac{{r}_{1}^{2}+{r}_{2}^{2}+{r}_{4}^{2}-{r}_{3}^{2}}{{2r}_{2}{r}_{4}}.$$

## Objective function and constraints formulation

In the path synthesis problem, we assume that we know a sequence of the desired points, which are in fact subsequent positions of the selected point of the mechanism, that is, point $\mathrm{E}$:

16 $$\left({x}_{\mathrm{P}1}^{Des},{y}_{\mathrm{P}1}^{Des}\right),\left({x}_{\mathrm{P}2}^{Des},{y}_{\mathrm{P}2}^{Des}\right),\dots ,\left({x}_{\mathrm{P}i}^{Des},{y}_{\mathrm{P}i}^{Des}\right),\dots ,\left({x}_{\mathrm{P}N}^{Des},{y}_{\mathrm{P}N}^{Des}\right) \mathrm\,{for}\, i=\mathrm{1,2},\dots ,N,$$

where $N$ denotes the number of desired points. The dimensional path synthesis is treated here as an optimization, in which the objective function to be minimized is presented in Sect. 3.1. Furthermore, it is subject to the constraints described in Sects. 3.2–3.4.

### Objective function

The objective function is defined here as follows:

17 $$f\left(\widehat{{\varvec{p}}}\right)=\sum_{i=1}^{N}\left[{\left({x}_{\mathrm{P}i}^{Des}-{x}_{\mathrm{P}i}^{Cal}\left(\widehat{{\varvec{p}}}\right)\right)}^{2}+{\left({y}_{\mathrm{P}i}^{Des}-{y}_{\mathrm{P}i}^{Cal}\left(\widehat{{\varvec{p}}}\right)\right)}^{2}\right],$$

where $\widehat{{\varvec{p}}}$ is the decision variables vector and $\left({x}_{\mathrm{P}i}^{Cal}\left(\widehat{{\varvec{p}}}\right),{y}_{\mathrm{P}i}^{Cal}\left(\widehat{{\varvec{p}}}\right)\right)$ is the $i$-th position of the point $\mathrm{P}$ calculated based on the given decision variables vector.

Furthermore, we can distinguish two cases of the path synthesis problem, namely, with and without prescribed timing. Thus, the vector $\widehat{{\varvec{p}}}$ of the decision variables can take the form

18 $$\widehat{{\varvec{p}}}=\left[{r}_{1},{r}_{2},{r}_{3},{r}_{4},{r}_{\mathrm{P}x},{r}_{\mathrm{P}y},{x}_{\mathrm{A}},{y}_{\mathrm{B}},{\theta }_{0}\right],$$

or

19 $$\widehat{{\varvec{p}}}=\left[{r}_{1},{r}_{2},{r}_{3},{r}_{4},{r}_{\mathrm{P}x},{r}_{\mathrm{P}y},{x}_{\mathrm{A}},{y}_{\mathrm{B}},{\theta }_{0},{\theta }_{2}^{1},{\theta }_{2}^{2},..,{\theta }_{2}^{N}\right],$$

for the path synthesis problems with and without prescribed timing, respectively.

### Constraint I: Grashof’s law

The first constraint is the well-known Grashof’s law. It requires that the sum of the shortest and longest bars of the mechanism is less than or equal to the sum of the lengths of the two other bars. The condition to meet Grashof’s criterion can be written in the form:

20 $$2\underset{}{\mathrm{min}}\left({r}_{1},{r}_{2},{r}_{3},{r}_{4}\right)+2\underset{}{\mathrm{max}}\left({r}_{1},{r}_{2},{r}_{3},{r}_{4}\right)<{r}_{1}+{r}_{2}+{r}_{3}+{r}_{4.}$$

Furthermore, to ensure the obtainment of a crank-rocker mechanism, an additional condition can be set in the following form:

21 $$\underset{}{\mathrm{min}}\left({r}_{1},{r}_{2},{r}_{3},{r}_{4}\right)=crank.$$

### Constraint II: ascending order of the input angles

It is also required that the input angles between the crank and the *x* axis satisfy the following condition:

22 $${\theta }_{2}^{1}<{\theta }_{2}^{2}<\dots <{\theta }_{2}^{N}.$$

This constraint refers to the path synthesis problem without prescribed timing.

### Constraint III: lower and upper limits of the design variables

All the design variables, from a practical standpoint and for the simplicity of the optimal solution search, need to be limited. Here, we denote the limits of these variables with the superscripts $L$ or $U$, for the lower and upper limits, respectively, for example, ${r}_{1}^{U}$ or ${\theta }_{2}^{1U}$.

### Objective function in the penalty function method

In the case when the penalty function method is applied, the objective function can be written in the following form:

23 $$f\left(\widehat{{\varvec{p}}}\right)=\sum_{i=1}^{N}\left[{\left({x}_{\mathrm{P}i}^{Des}-{x}_{\mathrm{P}i}^{Cal}\left(\widehat{{\varvec{p}}}\right)\right)}^{2}+{\left({y}_{\mathrm{P}i}^{Des}-{y}_{\mathrm{P}i}^{Cal}\left(\widehat{{\varvec{p}}}\right)\right)}^{2}\right]+{f}_{p},$$

where ${f}_{p}$ is the penalty coefficient, which takes a huge number (e.g., infinity if the programming language allows) if one the constraints described in Sects. 3.2–3.4 is unsatisfied. Otherwise, the penalty coefficient equals to zero.

## Path-repairing technique

In this paper, the path-repairing technique is applied to avoid inefficiently assigning large values to the objective function when the constraints are not met. This technique has been proposed by Bureerat and Sleesongsom [16, 33].

### Repairing of Grashof’s law

Grashof’s law is presented in Eq. (20). In many previous works, if the condition was not satisfied, the penalty function method was applied. Here, a repairing method is used. The whole repairing technique of Grashof’s law proposed in [16, 33] is presented in Table 1. At the beginning, four random numbers ${\delta }_{1},{\delta }_{2},{\delta }_{3},{\delta }_{4}$ are chosen. Then, in Step 2, it is ensured that the number ${\delta }_{3}$ is greater than the number ${\delta }_{1}$. After calculating the auxiliary variables ${S}_{i}$ ($i=\mathrm{1,2},\mathrm{3,4}$) in Step 3, the minimal value is ${S}_{2}$, which guarantees the satisfaction of condition (21), and the maximal value is ${S}_{1}$. Thus, the condition (20) takes the following form:

24 $${2S}_{1}+{2S}_{2}<{S}_{1}+{S}_{2}+{S}_{3}+{S}_{4},$$

which results in

25 $${S}_{1}+{S}_{2}<{S}_{3}+{S}_{4},$$

and

26 $${\delta }_{1}<{\delta }_{3},$$

which was guaranteed in Step 2. In Step 4, all the auxiliary variables ${S}_{i}$ ($i=\mathrm{1,2},\mathrm{3,4}$) are normalized if the maximal value is greater than 1. Finally, the resulting lengths of the bars are calculated based on the auxiliary variables ${S}_{i}$ ($i=\mathrm{1,2},\mathrm{3,4}$) in Step 5.

**Table 1:** Grashof’s law repairing algorithm

Input:	${{\varvec{r}}}_{1}^{{\varvec{o}}{\varvec{l}}{\varvec{d}}},{{\varvec{r}}}_{2}^{{\varvec{o}}{\varvec{l}}{\varvec{d}}},{{\varvec{r}}}_{3}^{{\varvec{o}}{\varvec{l}}{\varvec{d}}},{{\varvec{r}}}_{4}^{{\varvec{o}}{\varvec{l}}{\varvec{d}}}$ and the bounds: ${{\varvec{r}}}^{{\varvec{L}}}$,${{\varvec{r}}}^{{\varvec{U}}}.$
Step 1	Generate random numbers ${\delta }_{1},{\delta }_{2},{\delta }_{3},{\delta }_{4}$ in the range [0.0001,1]
Step 2	If ${\delta }_{1}>{\delta }_{3}$, swap their positions Else if ${\delta }_{1}={\delta }_{3}$, set ${\delta }_{3}={\delta }_{3}+0.0001$
Step 3	Calculate: ${S}_{2}={\delta }_{2}$, ${S}_{3}={\delta }_{2}+{\delta }_{3}$, ${S}_{4}={\delta }_{2}+{\delta }_{3}+{\delta }_{4}$, ${S}_{1}={\delta }_{1}+{\delta }_{2}+{\delta }_{3}+{\delta }_{4}.$
Step 4	If $\mathrm{max}\left({S}_{1},{S}_{2},{S}_{3},{S}_{4}\right)>1$, then for $i=\mathrm{1,2},\mathrm{3,4}$ compute: ${S}_{i}=\frac{{S}_{i}}{\mathrm{max}\left({S}_{1},{S}_{2},{S}_{3},{S}_{4}\right)}.$
Step 5	For $i=\mathrm{1,2},\mathrm{3,4}$ compute: ${r}_{i}^{new}={r}^{L}+\left({r}^{U}-{r}^{L}\right){S}_{i}.$
Output:	${r}_{1}^{new},{r}_{2}^{new},{r}_{3}^{new},{r}_{4}^{new}$

### Repairing the order of the input angles

In applying metaheuristics for the synthesis of four-bar mechanisms, it is very easy not to meet the ascending order of the input angles, here denoted as Constraint II, Eq. (22). In many previous studies in which metaheuristics were used for four-bar mechanism path synthesis, a penalty function was typically applied. In such a case, the objective function was set to a very large value if the criterion was not met. This led to a huge number of inefficient potential solutions. In most cases, this number is probably even greater than the number of efficient potential solutions. Therefore, in this study, a repairing technique is applied as proposed by Bureerat and Sleesongsom [16, 33]. The algorithm for repairing the order of the input angles consists of steps related to the generation of random numbers in the range [0.0001,1], scaling them and assigning the sums of these numbers to the input angles as the output from the repair algorithm. All the steps are presented in Table 2.

**Table 2:** Input angles repair algorithm

Input:	${{\varvec{\theta}}}_{2}^{1{\varvec{o}}{\varvec{l}}{\varvec{d}}},{{\varvec{\theta}}}_{2}^{2{\varvec{o}}{\varvec{l}}{\varvec{d}}},..,{{\varvec{\theta}}}_{2}^{{\varvec{N}}{\varvec{o}}{\varvec{l}}{\varvec{d}}}$
Step 1	Generate $N-1$ random numbers ${\alpha }_{1},{\alpha }_{2},..,{\alpha }_{N-1}$ in the range [0.0001,1]
Step 2	Scale the numbers ${\alpha }_{1},{\alpha }_{2},..,{\alpha }_{N-1}$ according to the following relation ($i=\mathrm{1,2},\dots ,N-1$): ${\alpha }_{i}^{scal}={\alpha }_{i}\frac{1.99\pi }{N-1}$
Step 3	Generate the new repaired input angles according to the following equation ($i=2,\dots ,N$): ${\theta }_{2}^{1new}={\theta }_{2}^{1old}$ ${\theta }_{2}^{i new}={\theta }_{2}^{i-1 new}+{\alpha }_{i-1}^{scal}$
Output:	${\theta }_{2}^{1new},{\theta }_{2}^{2new},..,{\theta }_{2}^{Nnew}$

## Virus optimization algorithm

The VOA was proposed by Liang and Juarez [20]. In this paper, it is applied in combination with the path-repairing technique for the path synthesis of the four-bar mechanism. The VOA is inspired by viruses, which attack living cells, and the potential solution is therefore represented by the location of a single virus. This metaheuristic is relatively easy to implement and has only some basic parameters, which is one of the main advantages of this algorithm. The parameters of the algorithm defined by the user are:

- maximal number of replications (*MNR*),
- number of initial solutions (*NIS*),
- number of strong viruses (*NSV*),
- growth rate of strong viruses (*GRSV*),
- growth rate of common viruses (*GRCV*).

**Fig. 2:**
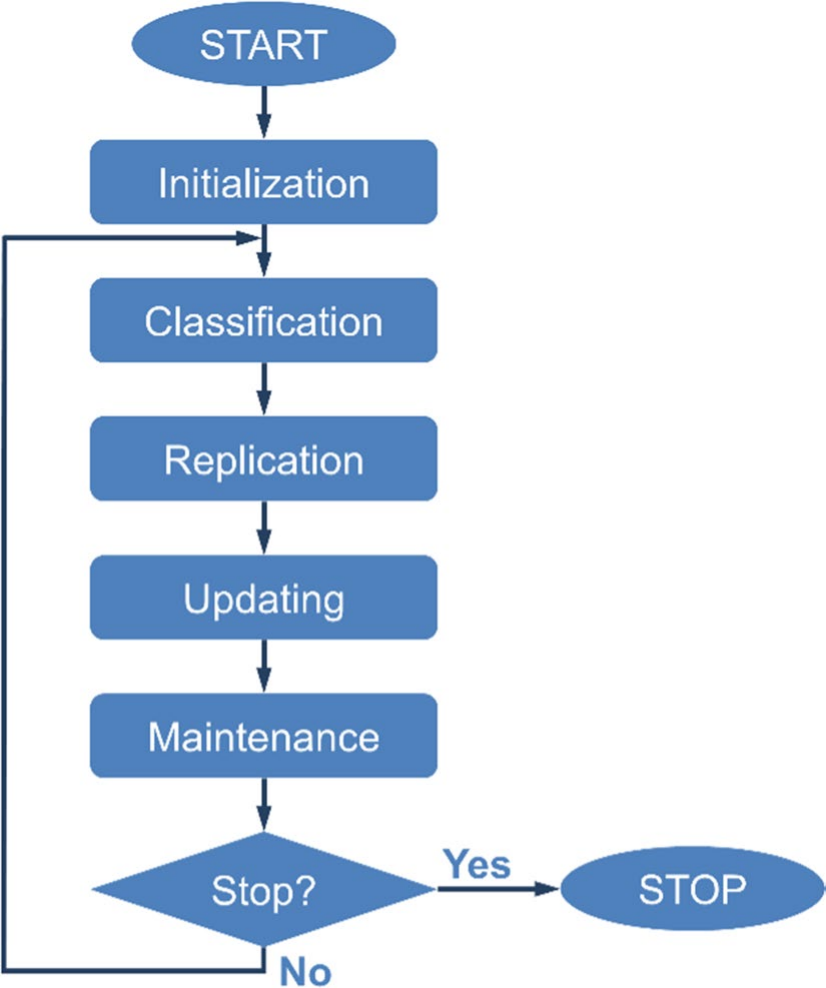
General flowchart of the virus optimization algorithm

The general flowchart of the VOA is presented in Fig. 2.

During the initialization step, an initial random population is generated. After that, the replication counter is set to 1.

At the classification stage, the viruses are classified into one of two groups, namely strong or common viruses, depending on the objective function values and the number of strong viruses. When a minimalization problem is considered, the *NSV* solutions, for which the minimal objective function is obtained, are set as strong viruses. The remaining solutions are common viruses.

The new viruses are generated during the replication stage. Each strong virus generates *GRSV* new viruses, and each common virus generates *GRCV* new viruses according to the following formulas:

27 $${NV}_{ij}={SV}_{ij}\pm \frac{\mathrm{rand}()}{intensity}{SV}_{ij},$$

28 $${NV}_{ij}={CV}_{ij}\pm \mathrm{rand}\left(\right){CV}_{ij},$$

for strong and common viruses, respectively. In the above equations, $NV$ denotes a new virus, $i$ is the member number in the population, while $j$ is the number of the design variable and $\mathrm{rand}()$ is the function generating random numbers in the range $\left[\mathrm{0,1}\right]$. The variable $intensity$ is initially set to 1 and can be modified in the next stage.

During the updating stage, the population convergence and algorithm performance are checked. If the average objective function value of the population has not improved, then the parameter $intensity$ is increased by one. This allows the exploitation of the areas occupied by the strong viruses.

At the next stage, called the maintenance mechanism or applying the antivirus, the number of viruses is reduced to *NIS* if the population size exceeds 1000. Furthermore, the number of viruses is reduced individually by considering the average objective function value until the number of eliminated viruses reaches the following value:

29 $$Amount=\mathrm{rand}\left(0,NV-NSV\right),$$

where $NV$ is the number of viruses (population size) and $\mathrm{rand}$ is the function generating the random integer number in the range $\left(0,NV-NSV\right)$.

In this study, the stopping criterion, or the maximal number of replications (*MNR*), is defined at the beginning of the optimization process, and after the number is reached, the algorithm stops. Otherwise, the procedure is repeated, starting with the classification stage.

A more detailed description of the algorithm can be found in [20].

## Results

The VOA was proposed for the optimization of the four-bar mechanism path generation problem. Two cases were considered: with (Case 1) and without (Case 2) prescribed timing. The first stage of the conducted research was to analyze the influence of the VOA parameters on the obtained values of the objective function, which were calculated according to formula (17). Moreover, the additional indicator, usually applied to determine performance accuracy in four-bar mechanism problems, was calculated using the following formula:

30 $$Error=\frac{1}{N}\sum_{i=1}^{N}\sqrt{{\left({x}_{\mathrm{P}i}^{Des}-{x}_{\mathrm{P}i}^{Cal}\left(\widehat{{\varvec{p}}}\right)\right)}^{2}+{\left({y}_{\mathrm{P}i}^{Des}-{y}_{\mathrm{P}i}^{Cal}\left(\widehat{{\varvec{p}}}\right)\right)}^{2}},$$

which is an average distance error between the desired points and the achieved points, obtained using the VOA.

In both cases (Case 1 and 2—with and without prescribed timing), the desired points were $\left({x}_{\mathrm{P}1}^{Des},{y}_{\mathrm{P}1}^{Des}\right)=\left(\mathrm{20,20}\right)$, $\left({x}_{\mathrm{P}2}^{Des},{y}_{\mathrm{P}2}^{Des}\right)=\left(\mathrm{20,25}\right)$, $\left({x}_{\mathrm{P}3}^{Des},{y}_{\mathrm{P}3}^{Des}\right)=\left(\mathrm{20,30}\right)$, $\left({x}_{\mathrm{P}4}^{Des},{y}_{\mathrm{P}4}^{Des}\right)=\left(\mathrm{20,35}\right)$, $\left({x}_{\mathrm{P}5}^{Des},{y}_{\mathrm{P}5}^{Des}\right)=\left(\mathrm{20,40}\right)$, $\left({x}_{\mathrm{P}6}^{Des},{y}_{\mathrm{P}6}^{Des}\right)=\left(\mathrm{20,45}\right)$. In Case 1, the input angles were taken as follows:

31 $${\theta }_{2}^{i}=\left[\frac{\pi }{24},\frac{\pi }{12},\frac{\pi }{8},\frac{\pi }{6},\frac{5\pi }{24},\frac{\pi }{4}\right].$$

The bounds of all design variables are summarized in Table 3.

**Table 3:** Bounds of the design variables

Design variables	Lower bound (*L*)	Upper bound (*U*)
${{\varvec{r}}}_{1}$, ${{\varvec{r}}}_{2}$, ${{\varvec{r}}}_{3}$, ${{\varvec{r}}}_{4}$ [mm]	5	60
${{\varvec{r}}}_{\mathbf{P}{\varvec{x}}}\boldsymbol{ }$[mm]	− 60	60
${{\varvec{r}}}_{\mathbf{P}{\varvec{y}}}\boldsymbol{ }$[mm]	− 60	60
${{\varvec{x}}}_{{\varvec{A}}}\boldsymbol{ }$[mm]	− 60	60
${{\varvec{y}}}_{{\varvec{A}}}\boldsymbol{ }$[mm]	− 60	60
${{\varvec{\theta}}}_{0}\boldsymbol{ }$[deg]	0	2π
${{\varvec{\theta}}}_{2}^{1}\boldsymbol{ },\boldsymbol{ }{{\varvec{\theta}}}_{2}^{2},{{\varvec{\theta}}}_{2}^{3},{{\varvec{\theta}}}_{2}^{4},{{\varvec{\theta}}}_{2}^{5},\boldsymbol{ }{{\varvec{\theta}}}_{2}^{6}\boldsymbol{ }$[deg]	0	2π

At the beginning, the path-repairing technique results were compared with the results of application the penalty method for Case 1 (Table 4). For both methods, the algorithms was performed 30 times. The minimal (Min) and maximal (Max), as well as mean (Mean) and standard deviation (Std) were calculated for the penalty and path-repairing technique, respectively. Based on the presented results, one can conclude that it is more beneficial to include in the algorithm the path-repairing technique than the penalty method. In case of the application of the penalty method, two runs were observed, for which the final solution was not obtained. Such a situation is not possible in the case of the path-repairing technique, because each solution which does not satisfy the constraints is modified in a way guaranteeing satisfaction of all the constraints. The path-repairing technique enables to significantly reduce the value of the objective function (mean value)—by 91%, and the results are more repeatable than in the case of penalty method (one can observe the values of the standard deviation in Table 4, column Std). Moreover, the obtained minimum value for the path-repairing technique is lower by over seven times than the best objective function value for the penalty method.

**Table 4:** Comparison of the obtained results for the penalty and path-repairing methods

Method	Min	Mean	Max	Std	Unsatisfied runs
Penalty	0.89	93.07	454.98	114.05	2
Path repairing	0.12	8.25	30.73	8.28	–

**Fig. 3:**
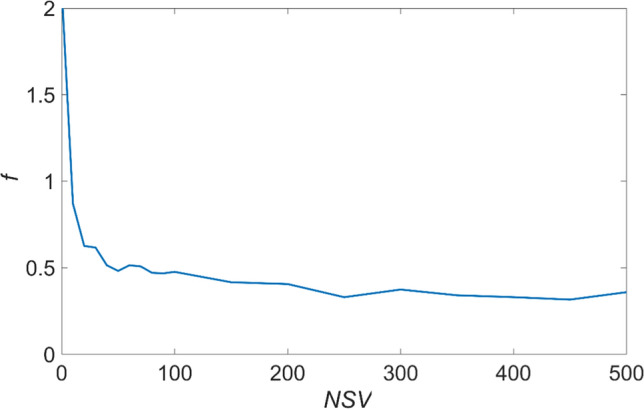
The mean value of the objective function in terms of the number of strong viruses (NSV)

The influence of three controllable VOA parameters were investigated: *NSV*, *GRSV*, and *GRCV*. The analysis for each parameter set was performed 30 times to average the results. The first parameter examined was the number of strong viruses (*NSV*). Values for this parameter were tested in the range 1–500 (see Table 4). The other parameters were as follows: *MNR* = 500, *NIS* = 500, *GRCV* = 5, and *GRSV* = 10. The values Min, Max, Mean and Std, presented in Table 5, relate to the values of the objective function achieved after *MNR* replications (iterations of the algorithm) in 30 trials for each parameter’s setup. Among those, the minimal (Min) and maximal (Max) value were highlighted, as well as mean (Mean) and standard deviation (Std). The change in the value of the objective function was observed. All these parameters decreased significantly when the *NSV* varied between 1 and 10 (see Fig. 3). When the *NSV* reached 300, no significant change in the value of the objective function was noticed. The chosen value for further experiments was equal to 300.

**Table 5:** Influence of the number of strong viruses (NSV) on the value of the objective function

*NSV*	VOA—Case 1			
	Min	Mean	Max	Std
1	0.4571	2.0000	6.1574	1.5075
10	0.1215	0.8711	4.4433	0.9858
20	0.0677	0.6264	1.2616	0.2724
30	0.0449	0.6174	2.0523	0.3875
40	0.0367	0.5148	1.6935	0.3279
50	0.0414	0.4823	0.7415	0.2169
60	0.1600	0.5150	1.3876	0.2444
70	0.1266	0.5082	1.3619	0.2734
80	0.0636	0.4712	0.7863	0.1799
90	0.0751	0.4682	1.3747	0.2882
100	0.1060	0.4768	0.7687	0.1751
150	0.0495	0.4168	0.7117	0.2002
200	0.1221	0.4064	0.7078	0.1947
250	0.0493	0.3304	0.6877	0.1916
300	0.0374	0.3746	0.6449	0.1818
350	0.0472	0.3413	0.7595	0.1918
400	0.0661	0.3306	0.6579	0.182
450	0.0692	0.3165	0.6868	0.1901
500	0.0784	0.3594	0.6663	0.1735

**Fig. 4:**
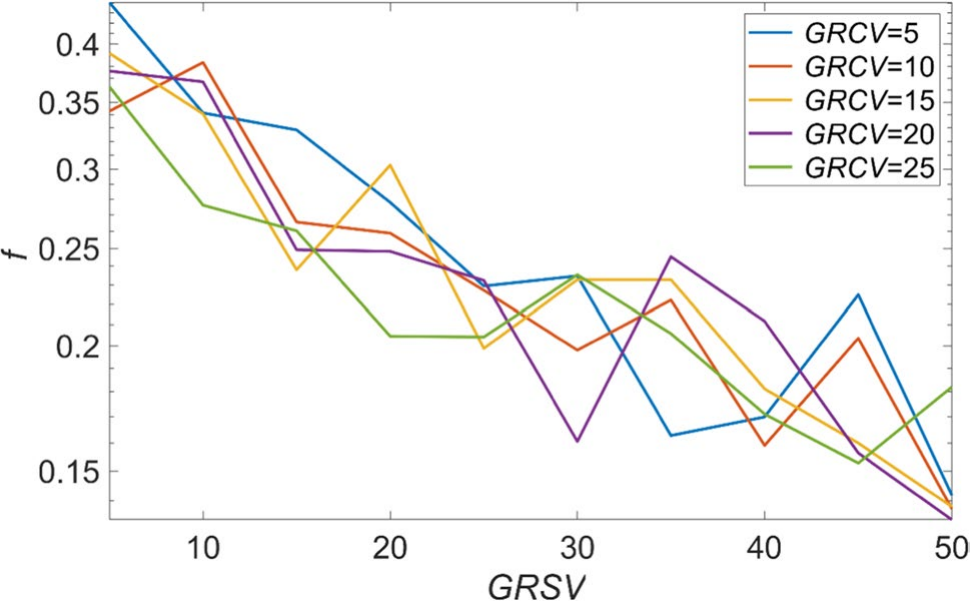
Relationship between the mean objective function value and GRSV for various GRCV values

The influence of the growth rate of strong viruses (*GRSV*) and growth rate of common viruses (*GRCV*) were investigated. The mean value of the objective function decreased with an increasing *GRSV* (see Fig. 4). The other parameters were as follows: *MNR* = 500, *NIS* = 500 and *NSV* = 300. The best result was obtained for a *GRCV* equal to 20 and a *GRSV* equal to 50.

**Fig. 5:**
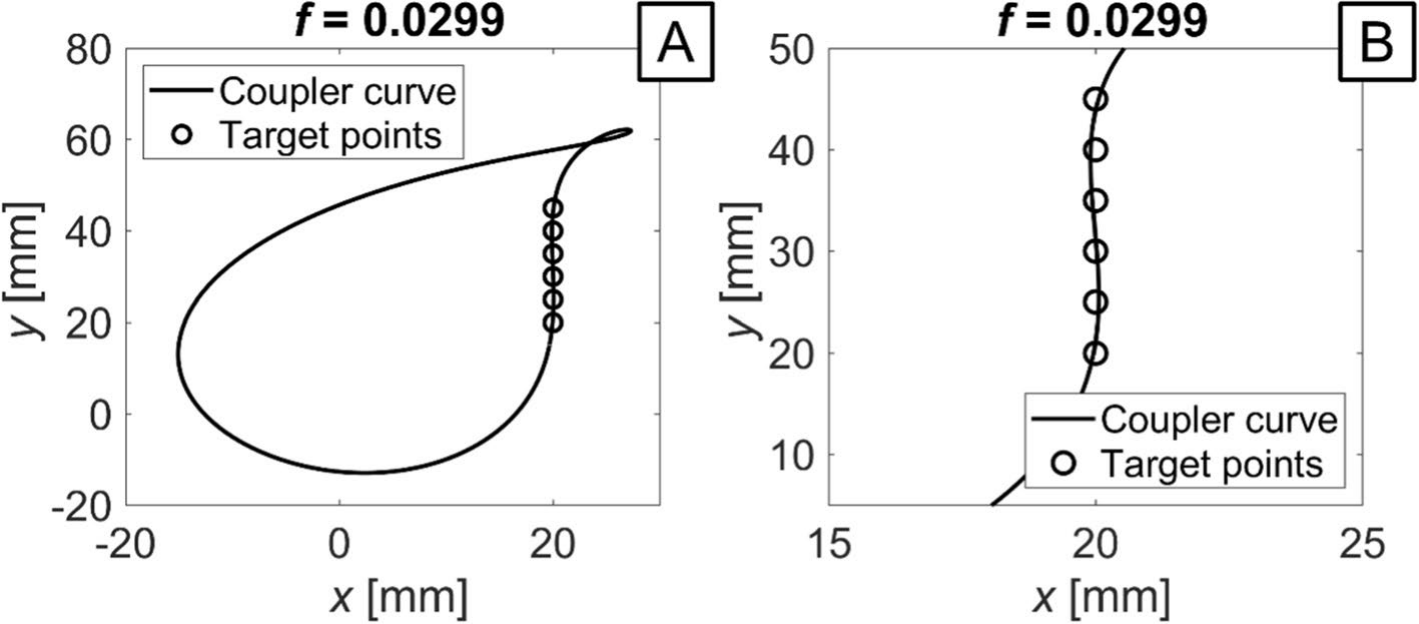
Coupler curve obtained for Min value of the final objective function in Case 1: **A** whole curve, **B** obtained path around desired points

**Fig. 6:**
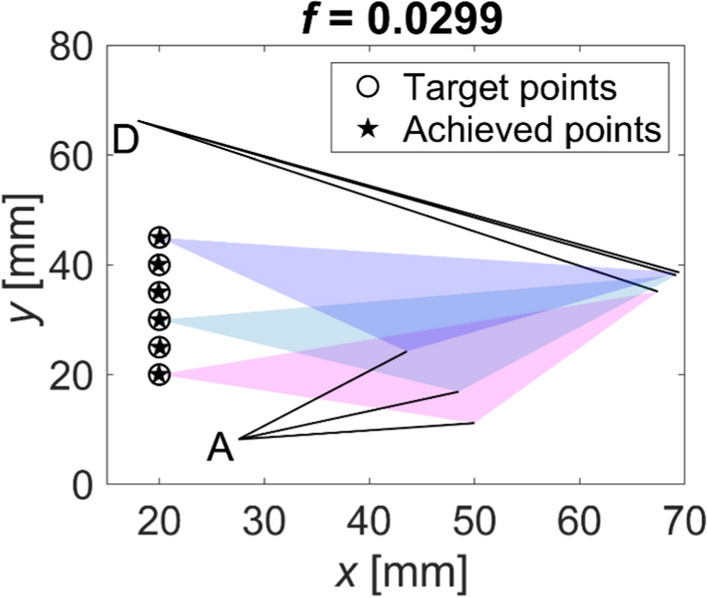
Mechanism obtained for Min value of the final fitness function in Case 1

**Fig. 7:**
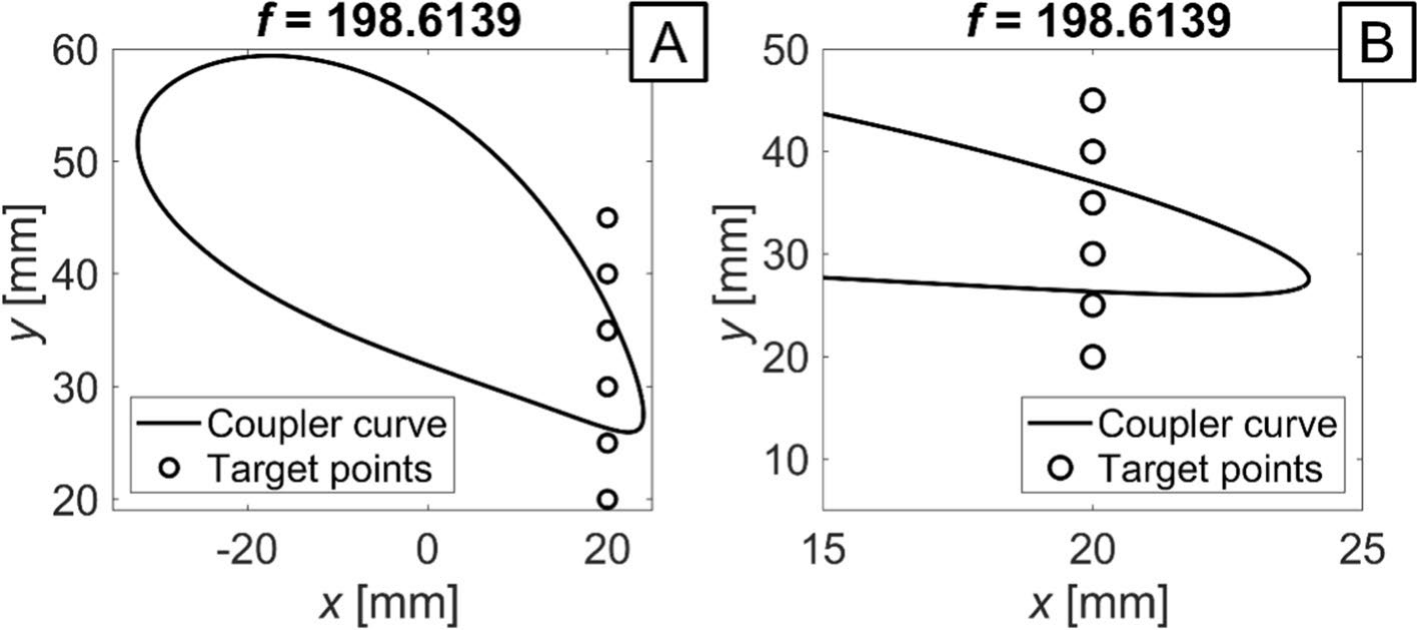
Coupler curve obtained at the beginning of the optimization process for the Min value of the final objective function in Case 1: **A** whole curve, **B** obtained path around desired points

**Fig. 8:**
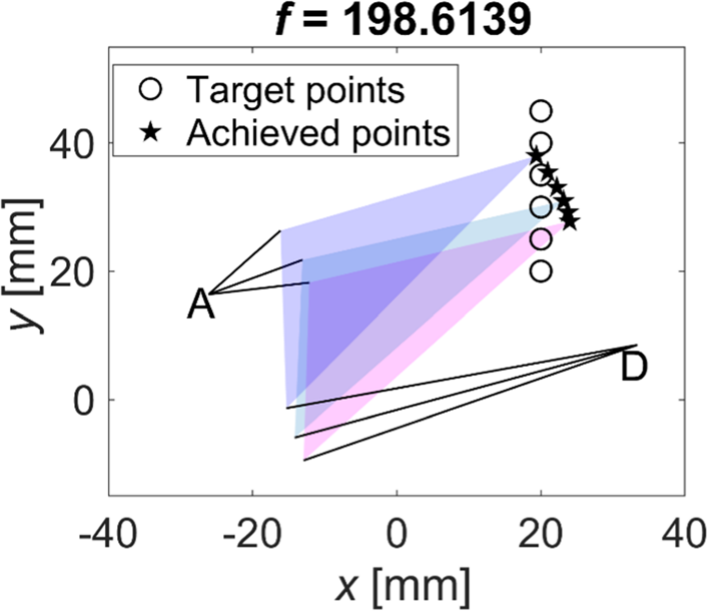
Mechanism obtained at the beginning of the optimization process for Min value of the final objective function

The conducted simulations allowed for the selection of the optimal parameters for the analyzed issue. The applied parameters and obtained results are shown in Table 6. The lowest value of the objective function (Min) was equal to 0.0299. The most precise obtained path for Case 1 is shown in Fig. 5. The ultimate mechanism resulting from the obtained design variables is depicted in Fig. 6. The results obtained during the first iteration of the algorithm differed significantly from the best achieved path (see Fig. 7). The mechanism after the first iteration is shown in Fig. 8. However, as the optimization process progressed, the obtained path improved.

**Table 6:** The results obtained for Case 1

Design variables	VOA—Case 1
	*MNR* = 500, *NIS* = 500, *GRCV* = 20, *GRSV* = 50
	*NSV* = 300
${{\varvec{r}}}_{1}\boldsymbol{ }$[mm]	58.8488
${{\varvec{r}}}_{2}\boldsymbol{ }$[mm]	22.6867
${{\varvec{r}}}_{3}\boldsymbol{ }$[mm]	29.6421
${{\varvec{r}}}_{4}\boldsymbol{ }$[mm]	58.4924
${{\varvec{r}}}_{\mathbf{P}{\varvec{x}}}\boldsymbol{ }$[mm]	-10.5067
${{\varvec{r}}}_{\mathbf{P}{\varvec{y}}}\boldsymbol{ }$[mm]	29.5122
${{\varvec{x}}}_{\mathbf{A}}\boldsymbol{ }$[mm]	27.5167
${{\varvec{y}}}_{\mathbf{A}}\boldsymbol{ }$[mm]	8.1954
${{\varvec{\theta}}}_{0}\boldsymbol{ }$[deg]	1.7345
Min	0.0299
Mean	0.1341
Max	0.6267
Std	0.1239
Error	0.0663

Using the parameters achieved during the analysis of Case 1, the process of finding the optimal solution for the four-bar mechanism for Case 2 was performed. The obtained results are shown in Table 7. In the previous case, with prescribed timing, the values $\theta_{2}^{1} {-}\theta_{2}^{6}$ were predefined, whereas in Case 2 those angles were fitted by the algorithm, as described in formula (19).

**Table 7:** The results obtained for Case 2

Design variables	VOA—Case 2
	*MNR* = 500, *NIS* = 500, *GRCV* = 20, *GRSV* = 50
	*NSV* = 300
${{\varvec{r}}}_{1}\boldsymbol{ }$[mm]	57.6925
${{\varvec{r}}}_{2}\boldsymbol{ }$[mm]	24.4863
${{\varvec{r}}}_{3}\boldsymbol{ }$[mm]	35.0356
${{\varvec{r}}}_{4}\boldsymbol{ }$[mm]	60.0000
${{\varvec{r}}}_{\mathbf{P}{\varvec{x}}}\boldsymbol{ }$[mm]	12.4974
${{\varvec{r}}}_{\mathbf{P}{\varvec{y}}}\boldsymbol{ }$[mm]	12.8388
${{\varvec{x}}}_{\mathbf{A}}\boldsymbol{ }$[mm]	13.3408
${{\varvec{y}}}_{\mathbf{A}}$[mm]	18.3209
${{\varvec{\theta}}}_{0}\boldsymbol{ }$[deg]	0.0611
${{\varvec{\theta}}}_{2}^{1}\boldsymbol{ }$[deg]	2.5156.10^–4^
${{\varvec{\theta}}}_{2}^{2}\boldsymbol{ }$[deg]	0.1331
${{\varvec{\theta}}}_{2}^{3}\boldsymbol{ }$[deg]	0.2539
${{\varvec{\theta}}}_{2}^{4}\boldsymbol{ }$[deg]	0.3728
${{\varvec{\theta}}}_{2}^{5}\boldsymbol{ }$[deg]	0.4960
${{\varvec{\theta}}}_{2}^{6}\boldsymbol{ }$[deg]	0.6319
Min	0.0024
Mean	0.0225
Max	0.1855
Std	0.0428
Error	0.0177

**Fig. 9:**
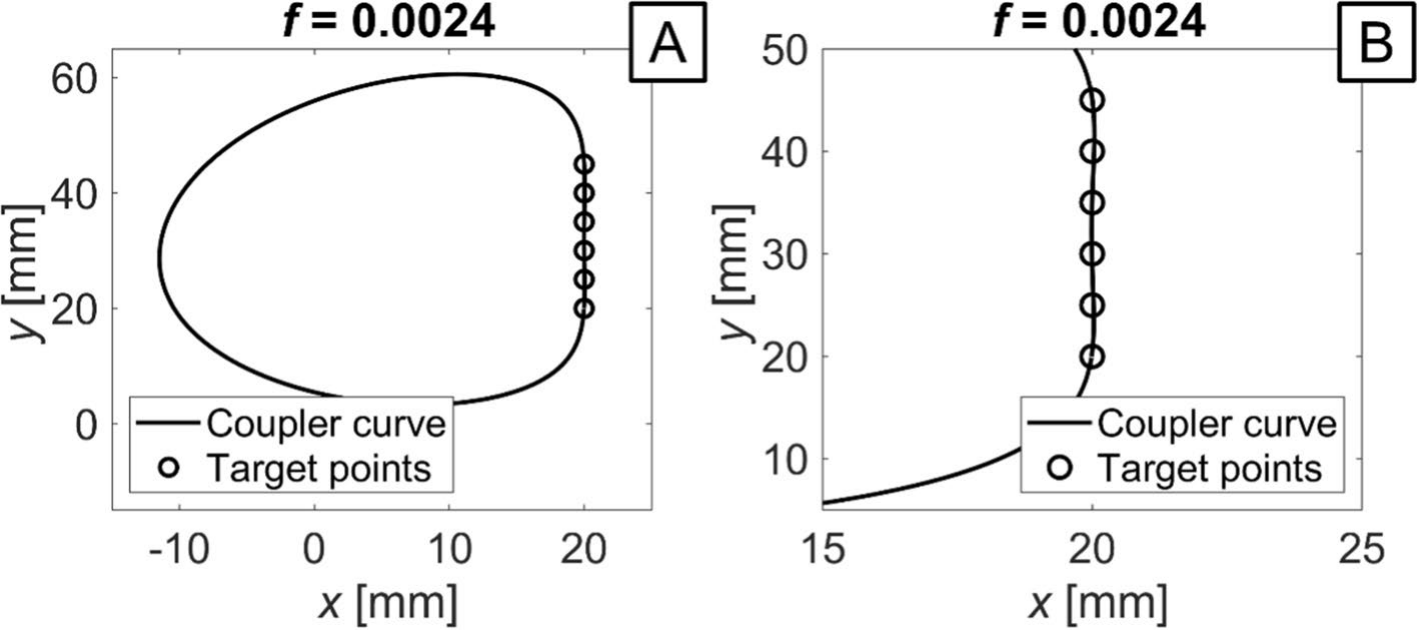
Coupler curve obtained for Min value of the final objective function in Case 2: **A** whole curve, **B** obtained path around desired points

**Fig. 10:**
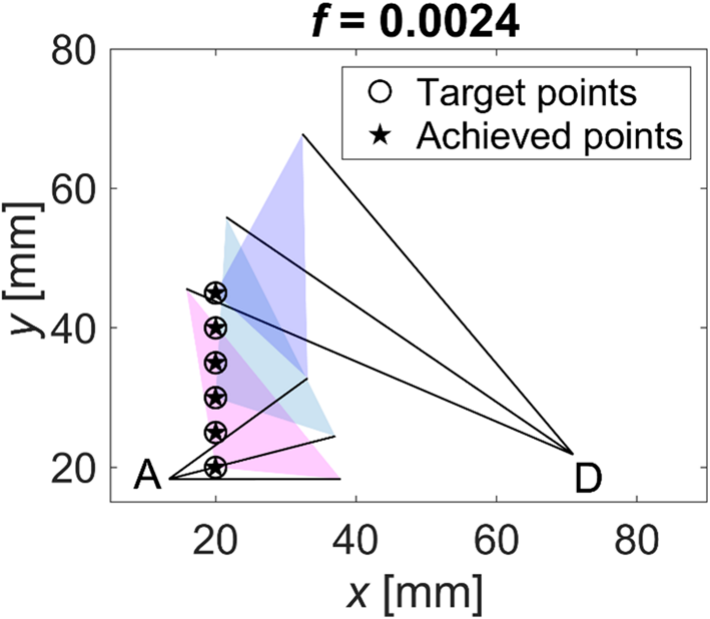
Mechanism obtained for Min value of the final objective function in Case 2

**Fig. 11:**
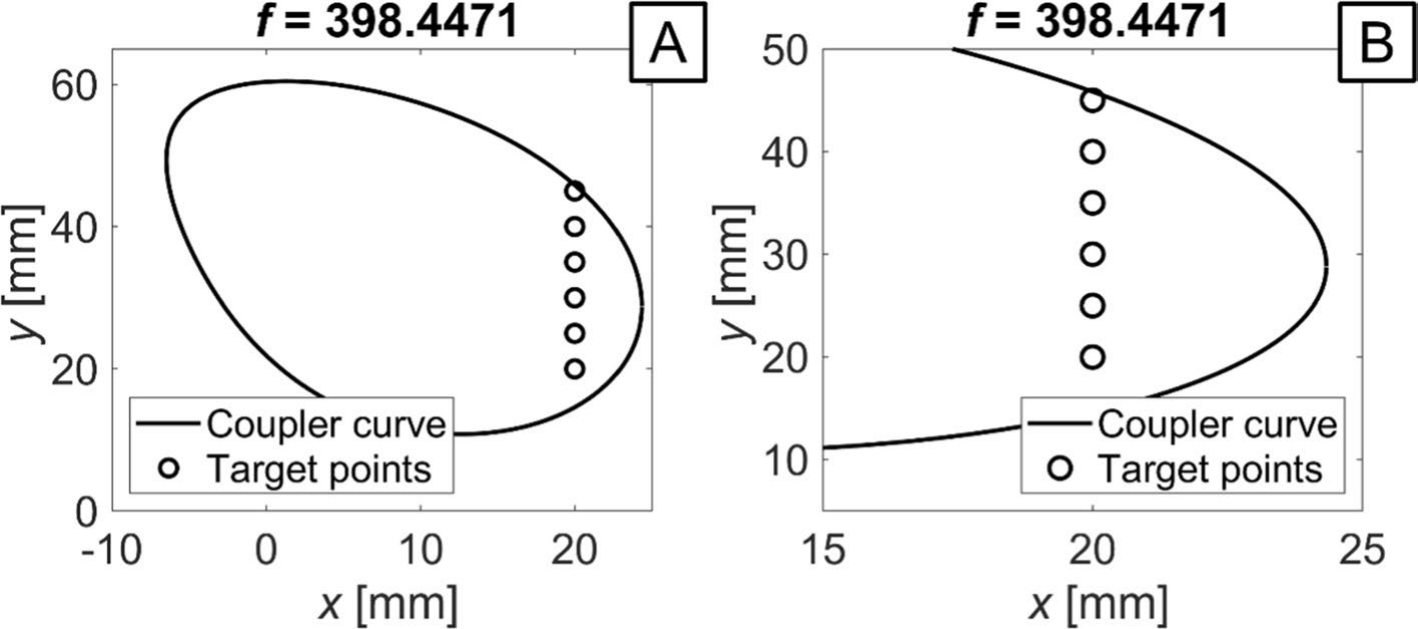
Coupler curve obtained at the beginning of the optimization process for Min value of the final objective function in Case 2: **A** whole curve, **B** obtained path around desired points

**Fig. 12:**
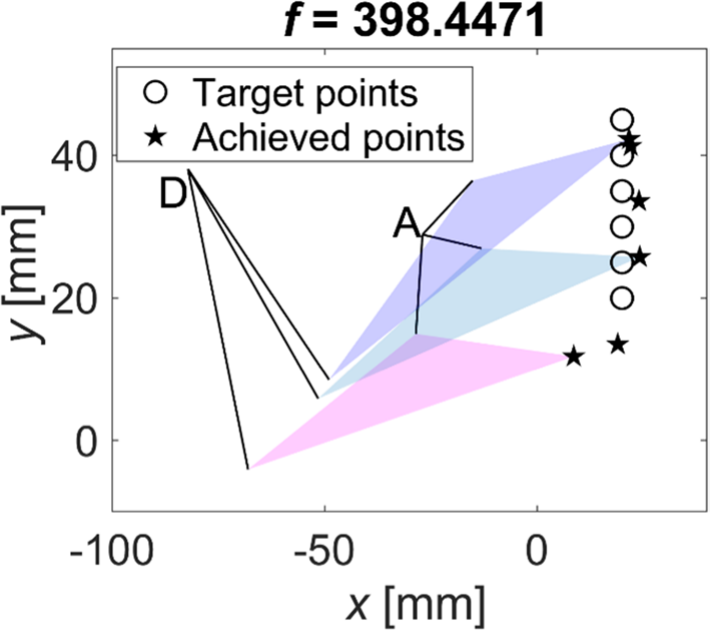
Mechanism obtained at the beginning of the optimization process for Min value of the final objective function in Case 2

Coupler curves, as well as mechanisms resulting from the optimization process for Case 2 (considering the Min value of the final objective function), are shown in Figs. 9 and 10. Furthermore, visualizations for the maximal objective function achieved in the same trial as the minimal (Min) are presented in Figs. 11 and 12. All the mechanisms satisfy the conditions previously described in formulas (20)–(22).

**Fig. 13:**
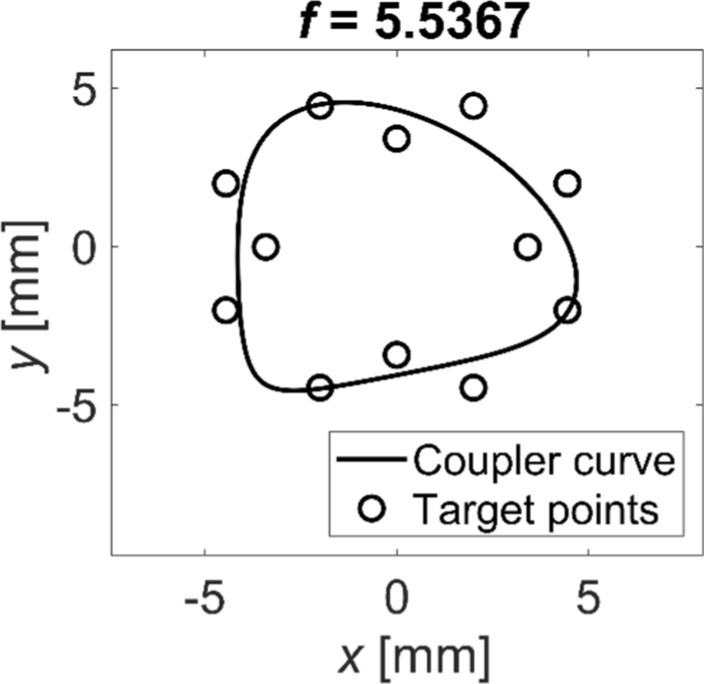
Coupler curve obtained for Min value of the final objective function of the four-circle waveform

**Fig. 14:**
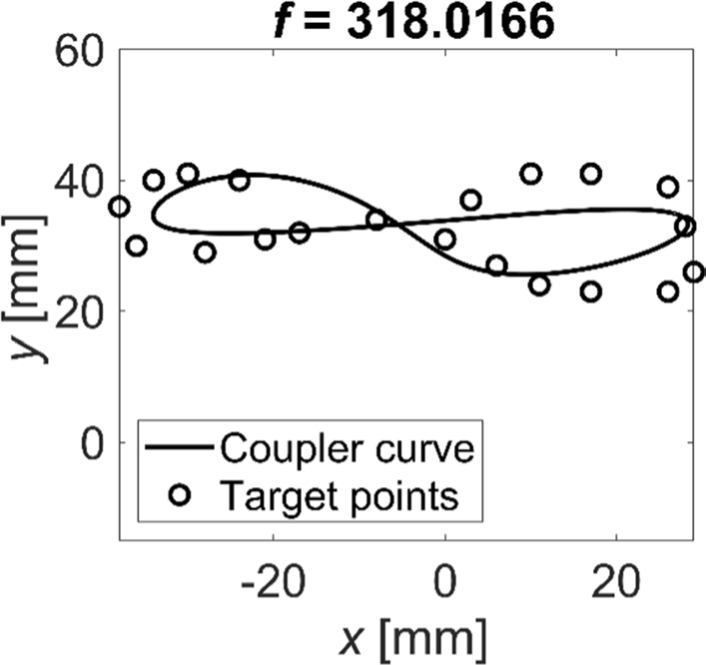
Coupler curve obtained for Min value of the final objective function of the infinity-type waveform

**Fig. 15:**
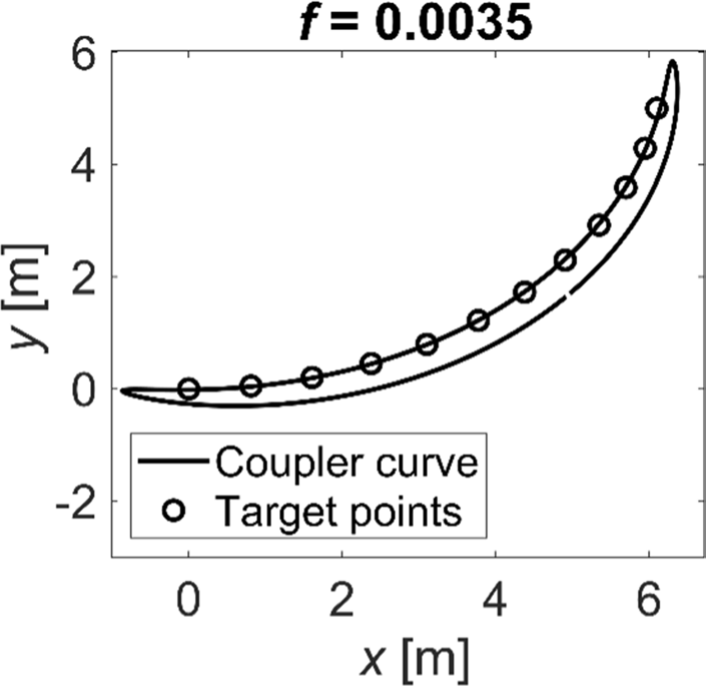
Coupler curve obtained for Min value of the final objective function of the landing gear waveform with lower *R* = − 100 and upper *R* = 100

**Fig. 16:**
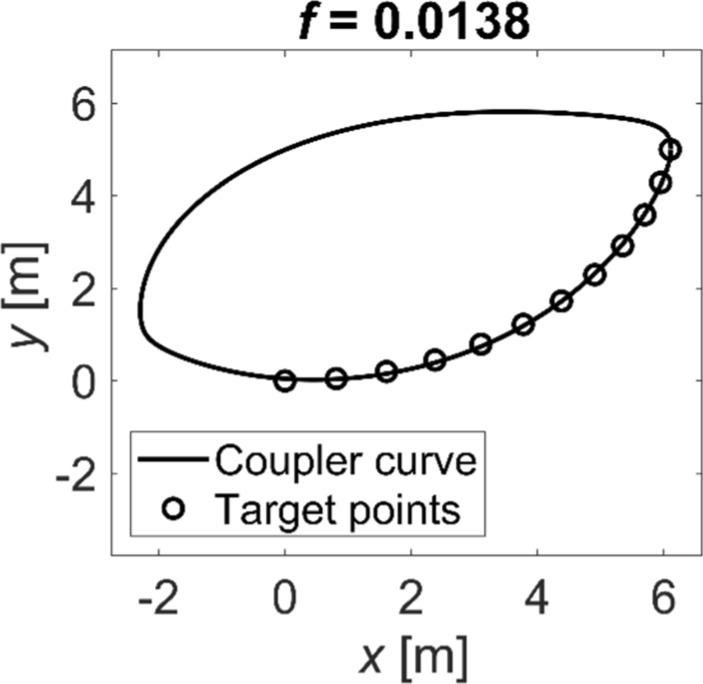
Coupler curve obtained for Min value of the final objective function of the landing gear waveform with lower *R* = − 20 and upper *R* = 20

**Fig. 17:**
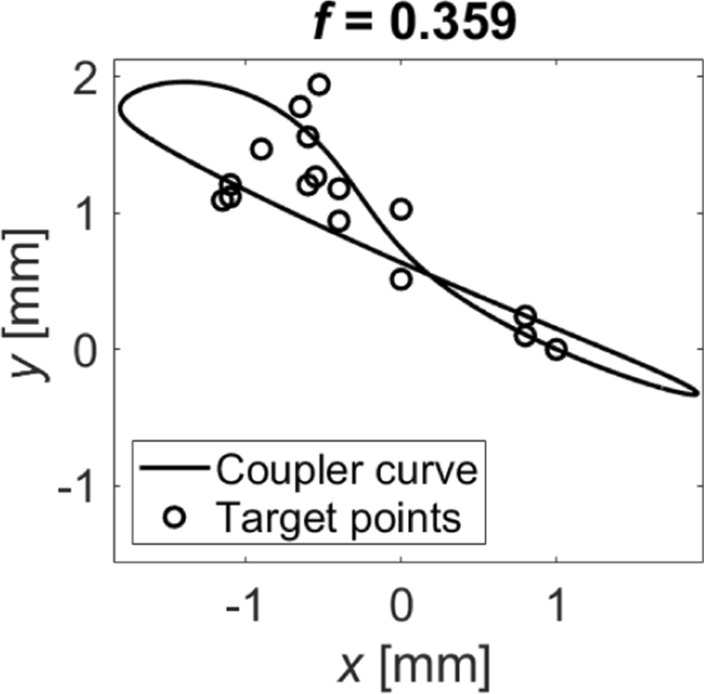
Coupler curve obtained for Min value of the final objective function of the symmetrical three-cusp curve

**Fig. 18:**
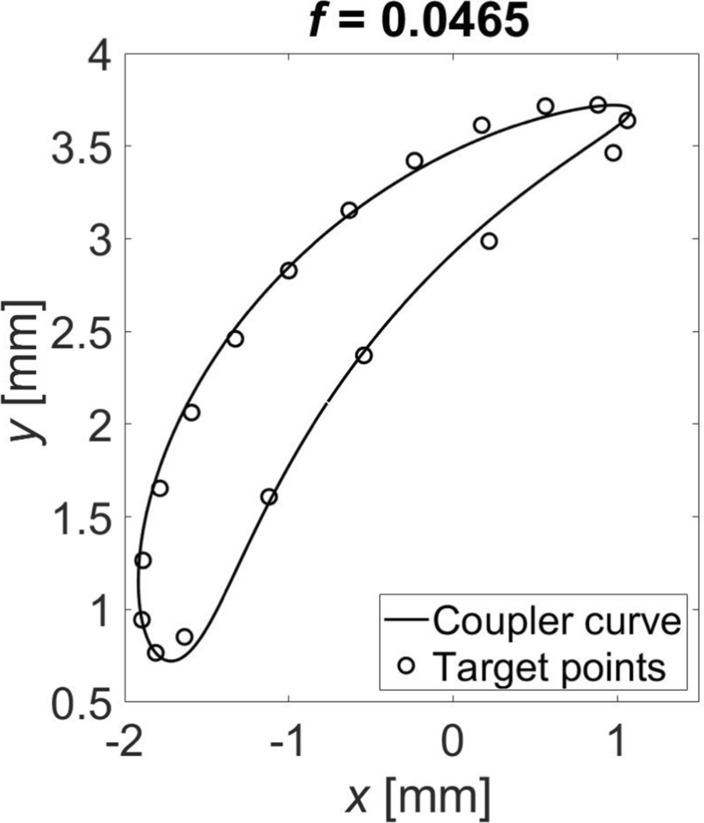
Coupler curve obtained for Min value of the final objective function of two-circular-arcs coupler curve

The analysis of VOA algorithm without prescribed timing was also conducted for more intricate paths [36–39]. Computation results are presented in Table 8. If the pattern was calculated with the use of optimization algorithm by another author, the objective function was also quoted. The obtained coupler curves are shown in Figs. 13, 14, 15, 16, 17, and 18. In comparison to the results obtained in the literature [36–39], one can notice that the results obtained in the current studies in some cases are better or worse than these presented in the papers [36–39]. For instance, in the case of landing gear the minimal objective function obtained in this study (0.0035) is greater than the objective function calculated for the solution presented in the paper (0.0003). However, the path obtained using the VOA with path-repairing technique is still satisfactory, see Fig. 15. For the case of two-circular-arcs coupler curve presented in the article [39], the objective function in this paper was equal to 0.2348, while in the current study the minimal value obtained was equal to 0.0465. The obtained path is also acceptable, see Fig. 18. Thus, it the results obtained using the proposed algorithm based on the VOA and path-repairing technique was better for this case.

**Table 8:** Minimal (Min), maximal (Max), mean values (Mean) and standard deviation (Std) of the objective function in 30 runs of the virus optimization algorithm for various path shapes without prescribed timing

Waveform	Min	Max	Mean	Std
Four-circle	5.5367	59.59107	7.9411	9.7949
Infinite-type path [37]	318.0166	1339.1748	597.6324	242.9498
Landing gear: *R* (− 100,100) [36]	0.0035	0.7852	0.2678	0.2807
Landing gear: *R* (− 20,20) [36]	0.0138	0.6342	0.2242	0.2219
Symmetrical three-cusp coupler curve [38]	0.3590	1.5216	0.7493	0.3715
Two-circular-arcs coupler curve [39]	0.0465	4.3223	0.7552	1.1284

## Conclusions

This study presented a successful combination of the path-repairing technique and virus optimization algorithm for the path synthesis of four-bar mechanisms. Based on the performed simulation, a set of an optimal parameters for the virus optimization algorithm for this problem could be defined.

The study found that an increase in the number of strong viruses (*NSV*) caused a decrease in the mean value of the final objective function until it reached a certain value, after which no significant change in algorithm accuracy was noticeable. Furthermore, as the growth rate of the strong viruses (*GRSV*) and the growth rate of the common viruses (*GRCV*) increased, the obtained path of the mechanism was more precise. This confirms the ease and intuitiveness of selecting these parameters, which is the most important advantage of the virus optimization algorithm over other metaheuristics.

Comparing the values of Min, Mean, Max, Std and Error obtained after performing a series of simulations, the results obtained in Case 2 were observed to be better than those obtained in Case 1. This was caused by the difference between these two problems, that is, the presence or absence of prescribed timing. However, despite the worse performance achieved in Case 1, the final values of the objective functions do not exclude the possibility of using the virus optimization algorithm to solve four-bar mechanism problems with predefined values of the input angles. Furthermore, the obtained results are still very precise.

Another important issue presented in this study was the application of the path-repairing technique, which guaranteed the efficient use of all possible solutions generated during the optimization process by means of the virus optimization algorithm.

The VOA algorithm was tested for various types of path. The most favorable results were obtained for the coupler curves, which were built on the basis of circular arcs. Also, lower values of objective functions were obtained for cases in which target points were placed only on part of all obtained by linkage path, allowing any necessary movements to occur outside of evaluated area.

## Data Availability

The data that support the findings of this study are available from the corresponding author upon request.
